# Toxic Metal Content in Deciduous Teeth: A Systematic Review

**DOI:** 10.3390/toxics13070556

**Published:** 2025-06-30

**Authors:** Ireneusz Zawiślak, Sylwia Kiryk, Jan Kiryk, Agnieszka Kotela, Julia Kensy, Mateusz Michalak, Jacek Matys, Maciej Dobrzyński

**Affiliations:** 1 Department of Human Nutrition, Faculty of Biotechnology and Food Science, Wroclaw University of Environmental and Life Sciences, Józefa Chełmońskiego 37, 51-630 Wroclaw, Poland; 2 Department of Pediatric Dentistry and Preclinical Dentistry, Wroclaw Medical University, Krakowska 26, 50-425 Wroclaw, Poland; s.roguzinska@gmail.com (S.K.); maciej.dobrzynski@umw.edu.pl (M.D.); 3 Dental Surgery Department, Wroclaw Medical University, Krakowska 26, 50-425 Wroclaw, Poland; jan.kiryk@umw.edu.pl; 4 Medical Center of Innovation, Wroclaw Medical University, Krakowska 26, 50-425 Wroclaw, Poland; kotela.agnieszka@gmail.com (A.K.); mateusz.michalak92@gmail.com (M.M.); 5 Faculty of Dentistry, Wroclaw Medical University, Krakowska 26, 50-425 Wroclaw, Poland; julia.kensy@student.umw.edu.pl

**Keywords:** heavy metals, toxic metals, deciduous teeth, milk teeth

## Abstract

Deciduous teeth accumulate toxic metals until fully mineralized, making them a stable biological matrix for assessing chronic exposure during fetal and early postnatal life. Their metal content is influenced by environmental factors (e.g., industrial areas, mining sites) and individual factors (e.g., maternal diet, early nutrition, passive smoking). The aim of this study was to evaluate the toxic metal content in deciduous teeth and to identify factors contributing to its accumulation, as well as possible health implications. A systematic review was conducted in accordance with the PRISMA guidelines and following the PICO framework. Quality assessment was assessed using the Joanna Briggs Institute (JBI) checklist for quasi-experimental studies. The literature search was carried out in the PubMed, Scopus, and Web of Science databases using the following keywords: deciduous, milk, primary, decidua, teeth, dentition, heavy metal, toxic metals. A total of 134 articles were initially identified, with 95 remaining after duplicate removal. After screening, 75 articles were excluded: 71 did not meet the inclusion criteria, 3 were not available in English, and 1 lacked full-text access. Ultimately, 20 studies were included in the review. Toxic metal concentrations were determined using various analytical techniques, mainly inductively coupled plasma mass spectrometry (ICP-MS) and atomic absorption spectrometry (AAS). Higher levels of metals, especially lead, were observed in the teeth of children residing in industrial areas, near mines, or in regions affected by armed conflict. Although two out of five studies indicated a possible link between fathers’ smoking habits and elevated lead concentrations, no definitive relationship was established between secondhand smoke exposure and the levels of lead and cadmium found in dental tissue. Similarly, no definitive relationship was identified between mercury and lead content and the prevalence of autism. However, lower manganese levels were associated with the presence of autistic traits, weaker verbal performance, and reduced memory capacity. In conclusion, deciduous teeth represent a valuable biological material for assessing chronic prenatal and early postnatal exposure to toxic metals, which may serve as a starting point for further research into diseases of unknown etiology, such as autism, and in the future may have clinical significance in their prevention and treatment. And it is also important for monitoring environmental pollution levels.

## 1. Introduction

Biological matrices such as blood, urine, hair, nails and teeth are commonly used in environmental studies [1]. Among these biological matrices, deciduous teeth are particularly noteworthy, as their mineralization process begins as early as 14 weeks of fetal life and continues until about 3 years of age [2,3]. The elemental composition of deciduous teeth reflects long-term individual exposure to toxic substances, such as toxic metals. During mineralization, toxic metals accumulate in dental tissues, making teeth a particularly useful biological material for assessing chronic exposure to toxic metals during both fetal and early postnatal life [4,5,6,7,8,9,10]. Moreover, due to the physiological process of replacing deciduous teeth with permanent ones, deciduous teeth can be collected in a non-invasive manner [11]. The use of deciduous teeth to assess exposure to toxic metals enables, among other things, the identification of sources of exposure and assessment of the effectiveness of preventive measures [5].

**Figure 1:**
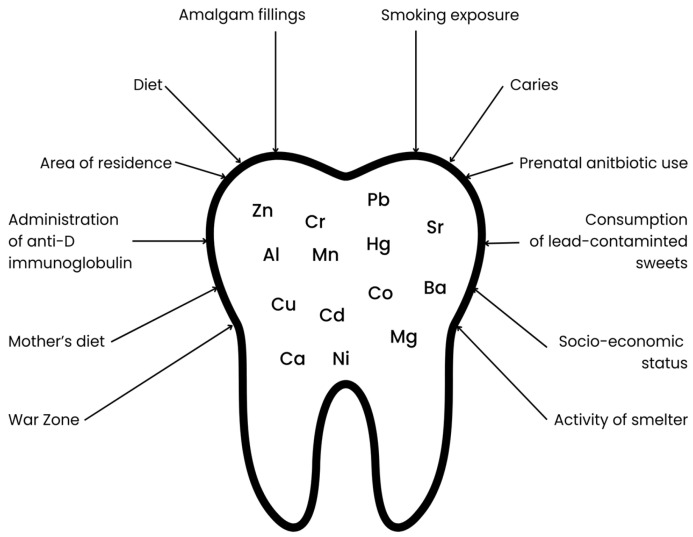
Factors influencing the content of selected elements in primary teeth.

The mineral composition of deciduous teeth is influenced by many factors, beginning with the mother’s lifestyle during pregnancy, the child’s living environment during early development, medications used by the mother during pregnancy, and medications taken by the child at an early age, as well as the child’s diet (see Figure 1). Increased retention of toxic metals is particularly promoted by maternal smoking during pregnancy, the child’s exposure to tobacco smoke, and living in industrial areas (near steel mills, factories, mines, or chemical plants) [12]. The use of antibiotics, which disrupt the gut microbiota, may contribute to increased accumulation and decreased excretion of toxic metals through the gastrointestinal tract [13,14]. Furthermore, diet is another critical factor influencing toxic metal content. A dietary pattern characterized by a low intake of calcium, iron, and zinc may contribute to an increased accumulation of toxic metals due to competition for common intestinal transporters. In addition, consumption of contaminated foods including fish and seafood (Hg), as well as rice and grain products (As), may further elevate toxic metal levels [15,16,17,18].

Of the many toxic substances and compounds found on Earth, those containing toxic metals have a particularly significant impact on human health and the environment. It is worth noting that toxic metals occur naturally in the Earth’s crust [19,20]. The greatest threats are posed by mercury, aluminum, chromium, arsenic, lead and cadmium, even in trace amounts. Their global distribution to the degree that threatens living organisms is primarily due to industry, agriculture, mining, and power plant operations [21,22,23]. They also enter the environment naturally, e.g., as a result of volcanic erosion [19]. Toxic metals present in water, soil and air lead to food contamination, which is how they enter the body [24,25]. They cause disorders affecting the nervous, digestive and cardiovascular systems and impair the functioning of internal organs such as the kidneys, lungs, liver and brain [19,26,27]. Toxic metals accumulate in the human body [28,29,30,31,32,33], which is why their effects can be both acute and chronic. Furthermore, toxic metals have been shown to have carcinogenic effects and have been classified as group 1 carcinogens [21,34,35].

This systematic review aims to assess the toxic metal content of primary teeth. There are a number of studies that have examined the content of toxic metals in primary teeth, taking into account various factors, but no review has been published to date. Examining the available literature revealed a need for a comprehensive review on this topic. Consolidating information on the accumulation of toxic metals in children’s teeth could raise awareness of global environmental pollution caused by toxic metals and encourage action to reduce human activities that contribute to environmental contamination.

## 2. Materials and Methods

### 2.1. Focused Question

The systematic review followed the PICO framework as follows: In the case of deciduous teeth (population), will exposure to environmental or lifestyle-related factors (investigated condition) result in differences in toxic metal content (outcome) compared to teeth from children not exposed to such factors (comparison condition)?

### 2.2. Protocol

**Figure 2:**
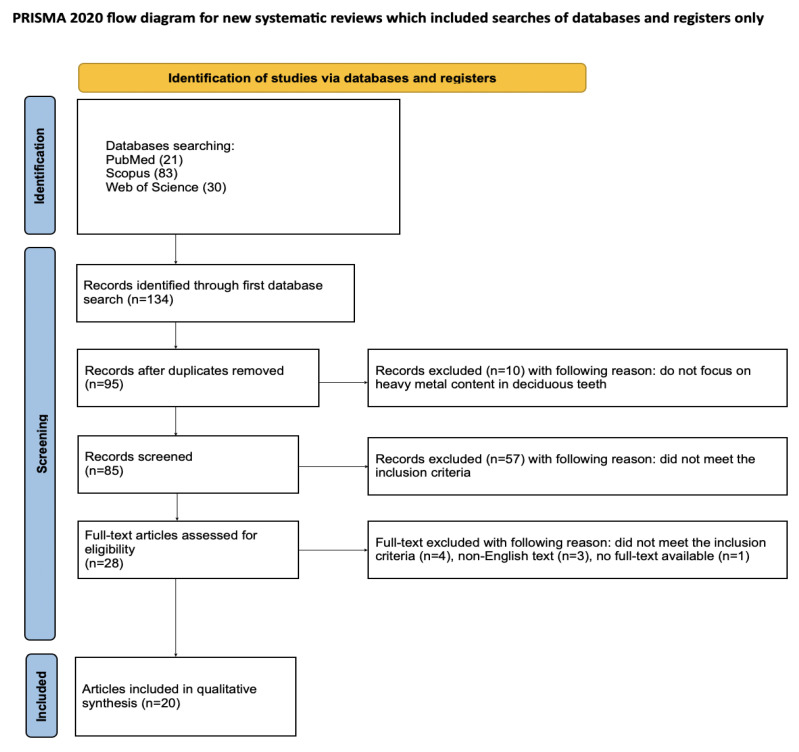
The PRISMA 2020 flow diagram [36].

Article selection for the systematic review followed a detailed methodology outlined through the PRISMA flow chart (Figure 2). PRISMA (Preferred Reporting Items for Systematic Reviews and Meta-Analyses) is a set of guidelines for reporting systematic literature reviews and meta-analyses, intended to ensure clarity and completeness of reports [36]. Registration of the systematic review was completed on the Open Science Framework at the specified link: https://osf.io/jywrn (accessed on 7 May 2025).

### 2.3. Eligibility Criteria

The following standards determined which investigations qualified for acceptance into the review:

- Studies conducted on deciduous teeth;
- Measurement of toxic metal concentrations;
- In vitro studies;
- Studies in English;
- Full-text articles.

The reviewers reached consensus on the following elimination standards:

- Studies focusing or permanent teeth or bones;
- Studies which did not examine the concentration of toxic metals;
- Non-English papers;
- Systematic review papers;
- Review articles;
- Not full-text accessible;
- Duplicated publications.

No restrictions were applied with regard to the year of publication.

### 2.4. Information Sources, Search Strategy, and Study Selection

In March 2025, a thorough literature search was conducted across PubMed, Scopus, and Web of Science (WoS) databases to identify studies that fulfilled the established inclusion criteria. The search targeted research related to concentration of toxic metals in deciduous teeth and was limited to titles and abstracts containing the keywords: deciduous OR milk OR primary OR decidua AND teeth OR dentition AND heavy metal OR toxic metals. All collected studies were assessed using predetermined selection criteria, and the final review contained solely those publications with obtainable full manuscripts.

### 2.5. Data Collection Process and Data Items

Six independent reviewers (I.Z., J.K., A.K., J.K., S.K. and M.M.) systematically selected studies that met the inclusion criteria. From each eligible article, data such as the first author’s name, year of publication, study design, article title, a type of toxic metal and its concentration were extracted. All information was organized and documented using a standardized Excel spreadsheet.

### 2.6. Risk of Bias and Quality Assessment

In the initial phase of study selection, each reviewer independently evaluated the titles and abstracts to minimize potential bias. The degree of agreement between reviewers was measured using Cohen’s kappa statistic. Any conflicts regarding the inclusion or exclusion of studies were addressed through discussion until consensus was reached among the authors.

### 2.7. Quality Assessment

The methodological quality of each included study was independently evaluated by two blinded reviewers (J.M. and M.D.) using the Joanna Briggs Institute (JBI) checklist for quasi-experimental studies (i.e., nonrandomized designs). This assessment tool consists of nine targeted questions designed to assess key aspects of study design and execution:

- Is it clear in the study what is the “cause” and what is the “effect”?
- Were the participants included in any similar comparisons?
- Were the participants included in any comparisons receiving similar treatment/care, other than the exposure or intervention of interest? Was there a control group?
- Were there multiple measurements of the outcome both before and after the intervention/exposure?
- Was a follow up completed, and if not, were differences between groups in terms of their follow up adequately described and analyzed? Were the outcomes of participants included in any comparisons measured in the same way?
- Were the outcomes measured in a reliable way?
- Was an appropriate statistical analysis used?

Each item was rated using one of four choices: “yes,” “no,” “not clear,” or “does not apply.” When evaluators provided different answers, they discussed the differences until they reached a shared conclusion. Inter-rater agreement was measured with Cohen’s kappa statistic, computed via MedCalc software (v23.1.7, MedCalc Software Ltd., Ostend, Belgium). The analysis yielded a kappa score of 0.86 (*p* < 0.001), reflecting strong agreement and high evaluator consistency.

## 3. Results

### 3.1. Study Selection

An initial database search of PubMed, Scopus, and WoS yielded 134 articles potentially relevant to the review. After removing duplicates, 95 articles were screened. After the initial screening of titles and abstracts, 67 articles that did not consider toxic metal content in deciduous teeth were excluded. It was not possible to access the full text of one article. Of the remaining 27 articles, 4 were excluded as they did not meet the inclusion criteria and 3 were excluded because full text was not available in English. Ultimately, a total of 20 articles were included in the qualitative synthesis of this review. The considerable heterogeneity among the included studies prevents the possibility of conducting a meta-analysis.

### 3.2. General Characteristics of the Included Studies

Most of the qualifying studies examined the content of more than one element [37,38,39,40,41,42,43,44,45,46,47,48,49,50,51,52,53], but only Friedman et al. did not decide to examine the amount of lead [54]. The level of lead in tissues was measured using various measurement techniques, including flame atomic absorption spectrometry (FAAS) [48], graphite furnace atomic absorption spectrometry (GFAAS) [37,38,43,52], inductively coupled plasma mass spectrometry (ICP MS) [40,41,42,44,45,46,47,51,55] and secondary-ion mass spectrometry (SIMS) [56]. Many authors have taken into account that the content of toxic metals in the body is influenced by the state of the environment in which the studied individuals live [39,41,43,49,50,51,52,54,55,56]; therefore, the place of conducting the studies is a significant heterogeneity of the studies. Thus, Twinnereim et al. [38] conducted studies on the teeth of children from Norway, three studies were based on samples from Iran [39,47,52], four from the USA [37,53,55,56], two from Mexico [45,56], two from Romania [41,51]; Amr et al. [42] studied children from Egypt, Gomes et al. [43] from Brazil, Yalcin et al. [46] from Turkey, Friedman et al. [54] from Italy, Savabiesfahani et al. [47] from Iraq and Lebanon, Fischer et al. [48] from Poland, and Bayo et al. [49,50] from Spain. Additional factors taken into account from the eligible studies were: presence of dental caries [38], distance from factories [39,53,54], socioeconomic status [39,49,50,52,53], serological conflict [37], mother’s diet during pregnancy [37,55], presence of amalgam fillings in both the mother during pregnancy and the examined child [37], child’s diet [52,56], and passive smoking [46,49,50,55] (see Table 1).

### 3.3. Main Study Outcomes

#### 3.3.1. Differences by Tooth Type and Presence of Caries

Tvinnereim et al. [38] demonstrated that deciduous teeth with caries exhibit higher concentrations of lead (Pb), mercury (Hg), and zinc (Zn) compared to healthy teeth. The study also highlighted a variation in metal accumulation by tooth type: incisors contained the highest levels of Pb, while molars had greater amounts of Hg and Zn. Moreover, the presence of dental roots was associated with increased Pb and Zn concentrations. These findings suggest that both the anatomical characteristics of the tooth and its pathological state influence its potential for metal accumulation. Contradictory results were reported by Motevaselian et al. [52], who found higher Pb levels in posterior teeth than in anterior ones, possibly due to differences in environmental or dietary exposure.

#### 3.3.2. Environmental Exposure: Industrial, Mining, and Conflict Zones

Children residing in polluted environments consistently showed higher concentrations of toxic metals in their deciduous teeth. For instance, Gomes et al. [43] reported significantly elevated Pb levels in children from industrial areas of Brazil, averaging 275.5 µg/g, compared to 183.1 µg/g in children from non-industrial regions. Similarly, Dinca et al. [51] found higher concentrations of copper, cadmium, chromium, and zinc in the teeth of children from the more polluted city of Slatina, Romania, relative to those from a less polluted city. In Iran, Nazemisalman et al. [39] observed elevated levels of Pb and Zn in children living near metal mines, regardless of tooth type or jaw location. Moreover, Savabieasfahani et al. [47] demonstrated that children living in war zones—specifically in Iraq and Iran—had high levels of Pb, particularly among those with congenital defects. These studies provide robust evidence that anthropogenic environmental contamination, whether due to industrial activities, mining operations, or armed conflict, contributes significantly to the bioaccumulation of toxic metals in primary dentition.

#### 3.3.3. Socioeconomic and Behavioral Factors

Socioeconomic status and individual behaviors were also found to influence metal concentrations in primary teeth. Bayo et al. [49,50] identified that children from lower-income families and older homes, especially in more polluted urban zones, had higher levels of Pb and Cd. Thumb sucking and nail biting were associated with increased Pb in upper jaw teeth. Furthermore, Cd concentrations were higher in non-carious teeth and in children not exposed to school-based fluoride programs. These findings suggest a complex interplay between social determinants and environmental exposure.

#### 3.3.4. Prenatal and Postnatal Exposure Patterns

Lead accumulation in deciduous teeth also reflects prenatal environmental exposure. Sitarik et al. [55] found that children with low birth weight and African American ethnicity had higher Pb levels in their teeth. A noteworthy finding was the correlation between prenatal Pb exposure and altered gut microbiota profiles, suggesting that even subclinical exposure may have systemic effects. Johnston et al. [53] confirmed that prenatal Pb concentrations in children’s teeth closely corresponded with soil contamination levels in areas surrounding a lead-acid battery recycling facility in California, underlining the value of teeth as biological matrices for assessing in utero exposure.

#### 3.3.5. Age and Dentition Differences

Several studies have shown that toxic metal concentrations in teeth decrease with age. Fischer et al. [48] and Bayo et al. [50] reported a decline in Pb and Cd levels as children grew older. Additionally, primary teeth differed significantly from permanent teeth in elemental composition. Tacail et al. [40] and Amr et al. [42] found lower levels of calcium, magnesium, strontium, and zinc in primary teeth, which may reflect both physiological differences in mineralization and varied timings of exposure.

#### 3.3.6. Associations with Neurodevelopmental Disorders

The evidence on the relationship between toxic metals and neurodevelopmental disorders is mixed. Adams et al. [37] reported elevated Hg levels in children with autism, though Pb and Zn did not differ significantly between groups. Abdullah et al. [44] did not find a clear association between autism and levels of Pb, Hg, or Mn. However, Yalcin et al. [46] identified significantly lower levels of strontium in children with autism and ADHD, suggesting a possible link between trace element deficiencies and neurodevelopmental outcomes. Additionally, Friedman et al. [54] associated elevated prenatal Mn levels with poorer cognitive and memory performance in adolescence, particularly among males (see Table 2).

### 3.4. Quality Assessment

For all of the 9 questions, 7 papers received a positive answer to 8 questions [37,38,41,43,46,51,53], 10 papers received a positive answer to 7 of them [39,42,44,45,48,49,50,52,54,55] and 3 received a positive answer to 6 questions [40,47,56] (see Table 3).

## 4. Discussion

Exposure to toxic metals can occur orally through the consumption of contaminated food or water [19,20], inhalation through inhaling dust containing metals in industrial areas or smoking [27], and percutaneously through skin contact with contaminated surfaces, cosmetics, paints, or metals in the work environment [20,57,58]. Regardless of the way they enter the body, their removal is not easy, which is why they accumulate in various tissues. The aim of this systematic review was to assess the presence of toxic metals in primary teeth. Of the 20 studies analyzed, 17 investigated more than one metal [37,38,39,40,41,42,46,48,49,50,51,52,53,56]. The most frequently studied elements were Pb [37,38,39,40,41,42,43,44,45,46,47,48,49,50,55,56], Zn [37,38,39,40,41,42,46,47], Cd [38,39,41,42,46,48,49,50], Cu [39,40,41,42,45,46,48] and Hg [37,38,44,46]. Particular attention was paid to analyzing metal concentrations in teeth [37,38,39,41,42,44,45,46,48,49,50,51,52,53,54,55,56], determining the origin of factors causing the presence of toxic metals in teeth [37,38,39,41,43,46,47,49,50,51,52,53,54,55,56], and investigating the effects of poisoning by these metals. The results indicate a strong relationship between environmental pollution and the accumulation of toxic metals in teeth. Increased concentrations have been noted among people living in industrial areas [41,43,51], mining areas [39], war zones [47] and those with a low socioeconomic status [49]. Elevated levels of toxic metals have also been found to cause neurological disorders [54], autism [37], lung function disorders [45] and low birth weight [55].

Twelve publications have demonstrated the impact of the living environment on the concentration of toxic metals in primary teeth [39,41,43,47,49,50,51,52,53,54,55,56]. Seven of these papers [39,41,43,47,51,53,54] focused on anthropogenic pollution from mines [39], smelters [41,54], warfare [47], a lead-acid battery recycling plant [53], and industrial areas [43,51]. All of the researchers involved in these studies found a positive correlation between environmental pollution and the concentration of toxic metals in primary teeth. In children from industrialized areas, the concentration of lead (Pb) was found to be more than 1.5 times higher than in children living in non-industrial areas, as demonstrated by Gomes et al. [43]. Furthermore, children in industrial areas were found to be more exposed to other toxic metals. Dinca et al. [51] noted increased concentrations of Cu, Cd, Cr and, in particular, Zn, while Nedelescu [41] proved that living in an area with smelter activity results in a Pb accumulation in primary teeth that is almost twice as high. In general, concentrations of other metals were found to be 60–95% higher, such as: Cd, Zn, Mn, Ni, Co and Cr. Despite the lack of a comparison with an unpolluted area, Nazemisalman et al. [39] believe that the concentrations of lead, cadmium, copper and zinc in the studied area are high and can only be attributed to the activity of lead and zinc mines. Savabieasfahani’s [47] observations suggest that warfare is also a significant source of exposure to toxic metals, particularly Pb. Similar concentrations of this metal have been found in the teeth of children in various locations around the world where wars are taking place.

The accumulation of toxic metals in primary teeth is not uniform. This issue was investigated in five studies [38,39,49,50,52]. Differences in metal accumulation were observed between incisors and molars, and, in one study [49], between the maxilla and mandible. Three out of four studies [38,49,50] observed that lead (Pb) accumulated mainly in upper arch teeth and incisors. According to two studies by Bayo et al. [49,50], cadmium (Cd) accumulated in greater amounts in incisors than in molars. In two other studies [38,39], no significant differences in the concentration of this metal were observed depending on the tooth. In contrast, according to Tvinnereim et al. [38], Hg and Zn accumulated more in posterior teeth. Mineralization of hard dental tissues is a process that, in the case of primary dentition, takes place from the fourth month of fetal life to one year after tooth eruption. The presence of toxic metals in teeth is associated with this process of mineralization. It is mainly then that elements can be incorporated into the tooth structure. Therefore, the presence of specific metals in specific teeth should be associated with exposure to toxic metals in the body during tooth mineralization. Differences in metal content have also been observed in primary and permanent teeth [40,42]. The lower content of Mg and Ca in primary teeth can be associated with physiologically lower mineralization; however, differences in the content of toxic metals should also be sought in the exposure to them during the mineralization period.

Most of the studies reviewed did not find a statistically significant correlation between lead and mercury content in deciduous teeth and the occurrence of neuropsychological disorders such as autism spectrum disorder or increased disruptive behavior [44,46]. However, a study by Yalçın et al. [46] showed that blood samples from children with autism spectrum disorder (ASD) had significantly higher concentrations of lead and cadmium, and lower concentrations of strontium, both in deciduous teeth and blood, compared to controls [46]. An exception to this trend was observed in the study by Adams et al. [37], which found statistically significantly higher mercury concentrations in the deciduous teeth of children with autism, while no such relationship was observed for lead. The discrepancy regarding the effect of mercury may be due to the preponderance of children with autism relative to the control group in the study by Adams et al. [37]. Additionally, the authors of the Yalçın et al. [46] study suggest that current exposure to toxic metals may exert a greater impact on the development of autism spectrum disorders than prenatal or early postnatal exposure. In addition, it was shown that the deciduous teeth of children with autism contained significantly lower concentrations of manganese compared to neurotypical children. Furthermore, it has been observed that children who had higher dentin manganese concentrations prenatally performed better in terms of cognitive function [5]. Most of the available studies focus on the toxic effects of excess manganese, neglecting the potential consequences of manganese deficiency [59,60]. Meanwhile, the work reviewed suggests that manganese deficiency during fetal development may adversely affect neurodevelopment, likely due to manganese’s role as a cofactor for numerous enzymes essential for normal body function [61]. The Savabieasfahani et al. [47] study also showed that the deciduous teeth of children with congenital malformations contained significantly higher lead concentrations compared to controls. Much previous work confirms the association between prenatal lead exposure and the occurrence of congenital malformations, particularly those involving the nervous, cardiovascular and skeletal systems [62,63,64,65,66]. This is probably related to the fact that lead readily crosses the placental barrier and exhibits teratogenic properties. The mechanisms of lead toxicity include the induction of oxidative stress, inhibition of key enzyme activity, mitochondrial damage and disruption of DNA methylation [67,68,69]. Another study showed that exposure to cadmium, manganese and, in the case of boys, lead at 12–15 weeks of fetal life significantly correlated with reduced lung function at 8–14 years of age [45]. This may be related to the overlap of two key stages of lung development at this time, the pseudomembranous phase and the tubular phase, during which the branching of terminal bronchioles into respiratory bronchioles and the formation of alveoli occur [70]. Exposure to toxic metals during this period may increase oxidative stress, resulting in abnormalities in lung development, which may have key implications for lung function [71]. Furthermore, it has been shown that lead content in deciduous teeth correlated significantly positively with Malassezia and Saccharomyces abundance and negatively with Candida, Penicillium and Aspergillus abundance in the gut microbiome [55]. Based on previous data, it can be speculated that higher lead concentrations favor the proliferation of Malassezia and Saccharomyces, while limiting the growth of Candida, Penicillium and Aspergillus through a mechanism of interspecies competition, thus influencing the relative composition of microorganisms in the gut [14,72].

There are several limitations to this systematic review that should be considered when interpreting the findings. The studies included employed a variety of analytical techniques—such as inductively coupled plasma mass spectrometry (ICP-MS), atomic absorption spectrometry (AAS), and laser ablation—each with differing levels of sensitivity and specificity, which hinders the comparability of results. Moreover, discrepancies in sample preparation methods (e.g., full mineralization vs. surface ablation) and small sample sizes in some studies may reduce statistical power and increase the risk of Type II error. The exclusion of non-English publications is another limitation, although it helped ensure full comprehension and accurate analysis of included studies. To improve future research, it is strongly recommended to adopt standardized protocols for sample preparation, metal quantification, and data reporting. Studies should aim to include large, well-characterized cohorts and report both relative and absolute concentrations of metals. Consistent testing and reporting standards would greatly enhance the comparability and reliability of findings across studies. In terms of biological mechanisms, several heavy metals such as lead, mercury, and cadmium are known neurotoxicants that can interfere with neurotransmitter systems, oxidative stress pathways, and calcium-dependent signaling during critical periods of brain development. For example, lead can disrupt synapse formation and plasticity, contributing to cognitive deficits and behavioral problems. Cadmium has been associated with impaired lung development and function through mechanisms involving inflammation and oxidative damage. These pathways underscore the relevance of assessing metal accumulation in deciduous teeth as an early indicator of potential developmental risks. Future research could benefit from incorporating advanced statistical approaches such as Monte Carlo simulations to better estimate individual and population-level risk. These tools allow for the integration of variability and uncertainty across multiple exposure factors, enhancing the reliability of risk assessments based on toxic metal concentrations in primary teeth.

## 5. Conclusions

In conclusion, deciduous teeth can be used as a reliable and non-invasive biological matrix reflecting chronic exposure to toxic metals during both the prenatal and early postnatal periods. Their mineralized structure allows for the long-term retention of toxic metals, providing a valuable archive of environmental exposure during critical stages of development. The results of this review highlight the significant influence of environmental and lifestyle-related factors, such as industrial pollution and proximity to mining areas, on the accumulation of toxic metals in deciduous teeth. While associations with specific health outcomes, including autism, remain inconclusive, observed correlations with neurodevelopmental disorders, particularly those linked to manganese deficiency, underscore the need for further research. Monitoring the elemental composition of deciduous teeth may support the early detection of environmental hazards and contribute to the development of public health strategies aimed at reducing exposure to toxic metals among vulnerable populations.

## Figures and Tables

**Table 1:** General characteristics of included studies.

Study	Aim of the Study	Material and Methods	Results	Conclusions
Tvinnereim et al. [38]	To assess how tooth type, presence of roots, and caries in deciduous teeth affect the concentrations of lead (Pb), cadmium (Cd), mercury (Hg), and zinc (Zn).	1271 deciduous teeth were analyzed (without fillings) for metal content using atomic absorption spectrometry (AAS). Sample sizes varied by element: 1271 for Pb, 554 for Hg, 1235 for Cd, and 1263 for Zn. Different AAS techniques were employed: flame technique (FAAS) for Zn, graphite furnace technique (GFAAS) for Pb and Cd, and cold vapor technique (CV-AAS) for Hg.	Carious teeth showed elevated Pb, Hg, and Zn (particularly in canines/molars). Teeth with roots contained higher Pb and Zn (especially in canines/molars). Pb was highest in incisors while Hg and Zn peaked in molars. Cd showed no significant differences between groups. Positive correlations existed between Pb and Cd/Hg/Zn, while Zn–Hg correlation appeared only in carious teeth.	The elemental composition depends on the tooth type, as well as the presence of roots and dental caries.
Nazemisalman et al. [39]	Evaluating Zn, Cu, Cd, and Pb concentrations in children living near lead and zinc mining areas in Zanjan, Iran.	Caries tissues were removed from 42 deciduous teeth using endodontic files. Teeth were cleaned with double-distilled water, 10% HNO_3_, and 30% H_2_O_2_. Elements were analyzed using polarographic system with anodic stripping voltammetry (ASV).	Toxic metal concentrations were elevated in teeth of children living near lead and zinc mines. No significant differences were found based on tooth type, jaw, or age for Pb, Cd, Cu, and Zn levels. The only significant variation was in zinc levels between males and females.	Deciduous teeth have proven to be effective biological matrices, reflecting severe environmental pollution through elevated levels of toxic metals.
Tacail et al. [40]	To improve trace element distribution analysis in tooth enamel for toxic metal exposure assessment and dietary reconstruction, while developing an LA-ICPMS sampling framework.	The study material consisted of 10 deciduous teeth and 12 permanent teeth. The elemental content was determined using inductively coupled plasma mass spectrometry with laser ablation (LA-ICPMS).	Deciduous teeth have lower Ca than permanent teeth with no spatial variation. Pb/Ca remains constant across enamel. Cu/Ca and Ni/Ca differentiate deciduous from permanent enamel. Permanent teeth show higher Zn/Ca (increasing at surface) and elevated Sr/Ca and Ba/Ca (decreasing outward)—gradients absent in deciduous enamel.	For reliable elemental analysis of life-history exposure, laser ablation should target the enamel–dentine junction where the most complete chemical record exists. Uniform Ca levels within teeth indicate homogeneous enamel maturation before eruption.
Adams et al. [37]	Assessment of mercury, lead, and zinc levels in deciduous teeth of children with autism compared with a control group.	Deciduous teeth from 15 children with autism and 10 neurotypical children were analyzed using atomic absorption spectrometry: Zn via flame AAS, Pb via graphite furnace AAS, and Hg via cold vapor AAS.	Mercury levels in teeth from children with autism were more than twice the average of the control group. Lead and zinc concentrations showed no statistically significant differences between the groups.	Children with autism showed elevated mercury exposure during prenatal and early postnatal development. Oral antibiotic use disrupts gut microbiome, potentially reducing mercury excretion and increasing absorption.
Ericson [56]	Assessment of Pb and Ca content in deciduous tooth enamel for use as a temporal biological matrix in evaluating prenatal and postnatal exposure.	Analysis of polished, carbon-coated longitudinal sections of enamel from deciduous mandibular central incisors from 3 children (including one with multiple lead exposure sources). Lead and calcium contents were measured using secondary-ion mass spectrometry (SIMS).	Total Pb content ranged from 1.1 to 3.7 ppm. Lead body burden (BBL) correlates with environmental lead exposure, where total lead can be estimated by multiplying 208 Pb by 1.9 (208 Pb represents 52.4% of total lead) to assess cumulative prenatal and postnatal exposure.	Lead content variation across samples indicates differences in environmental conditions or individual exposure levels. Using 208 Pb measurements provides a method for evaluating cumulative lead exposure throughout development. Multiplying 208 Pb by 1.9 converts to total lead concentration, enabling estimation of overall exposure.
Nedelescu [41]	Assessment of toxic metal content in primary teeth and hair of children from polluted industrialized areas.	The primary teeth and hair of 6 children aged 5–12 were examined. The samples were assessed for metal content: Pb, Zn, Cr, Mn, Cu, Cd, Ni using an inductively coupled mass spectrometer.	The content of toxic metals in the teeth of children from the studied area (Copsa Mica-Ruminia) was 60–95% higher than in children from unpolluted areas. Only the level of Pb in teeth was statistically significantly overestimated.	Toxic metals accumulate in teeth and hair. Their content in these tissues can be an indicator of pollution.
Sitarik [55]	To investigate the association between prenatal and postnatal lead levels and intestinal bacterial and fungal microflora in the first year of life.	Stool samples were collected from children aged 1–6 months to examine microflora. Later, they collected primary teeth from the same children after exfoliation and analyzed lead content above the neonatal line.	Higher lead levels were found in black, urban, low-birth-weight babies. Lower levels were found in babies whose mothers had contact with pets in the third trimester of pregnancy.	Higher lead levels in parts of primary teeth that formed during the second trimester of pregnancy were associated with lower abundance of Candida and Aspergillus and higher abundance of Malassezia and Saccharomyces.
Amr [42]	Comparison of the concentrations of toxic and essential elements in primary and permanent teeth collected in the city of Al-Kanayat, Egypt.	Primary teeth from 64 children were ground and their elemental content was analyzed using inductively coupled plasma mass spectrometry. The amounts obtained were compared with those in permanent teeth.	The mean concentrations of Na, Mg, Al, Fe, Ni, Cu, Sr, Cd, Ba, Pb and U were higher in permanent teeth, whereas concentrations of Mn, Co, As, Se, Mo and Bi were higher in primary teeth.	The concentration of toxic elements is higher in permanent teeth than in primary teeth.
Gomes [43]	To assess lead content in primary teeth surface enamel of preschool children and compare results based on their different living environments.	Enamel biopsies were taken from primary teeth of 329 children (some from industrial areas) and lead content was measured using graphite furnace atomic absorption spectrometry.	Lead levels in children’s tooth enamel showed 68.6% of children from non-industrial areas and 56.1% from industrial areas had 100–200 μg/g, while 44.2% from non-industrial areas and 28.8% from industrial areas had <100 μg/g.	Lead concentrations in enamel biopsies taken from children living in industrial areas were significantly higher than in biopsies from children living far from industry.
Abdullah [44]	To measure lead, mercury and manganese concentrations in primary teeth enamel of children with autism spectrum disorder and disruptive behavior, comparing results with normally developing children.	The content of elements in the enamel from the prenatal and postnatal periods was determined separately using laser ablation inductively coupled plasma mass spectrometry.	Children with autism spectrum disorder had lower levels of manganese in their postnatal enamel than healthy children and this is the only significant difference in the compared groups.	There is no association between exposure to toxic metals in very early life and the etiology of autism spectrum disorders and disruptive behavior.
Rosa [45]	To investigate the relationship between elevated concentrations of toxic metals in primary teeth and a deterioration in lung function in childhood.	The content of As, Cd, Co, Cu, Mn, Ni and Pb in the dentin of primary teeth was examined by laser ablation inductively coupled plasma mass spectrometry in children with a medical history of pulmonary dysfunction and compared with the results of healthy children.	An association was found between increased levels of cadmium and manganese in the period 12 to 14 weeks before birth with impaired lung function at 8–14 years of age.	Prenatal exposure to metals is associated with impaired lung function in childhood.
Yalcin [46]	Comparison of the content of Ca, Mg, Zn, Cd, Hg, Pb, Cu, Cr, Fe, Mn, Mo, P, Sr in the dentin of primary teeth of children with autism spectrum disorder and ADHD with the results obtained in normally developing children.	The content of elements in the dentine of primary teeth in children with ASD or ADHD confirmed by a psychiatrist and in healthy children was examined. Inductively coupled plasma mass spectrometry was used for this purpose.	Sick children had a significantly lower Sr level and Sr/Ca ratio.	Children with ASD and ADHD have significantly lower Sr levels in the dentin of primary teeth.
Friedman [54]	To investigate the association between early Mn exposure and adolescent neurodevelopment.	Primary teeth dentin manganese content was measured using inductively coupled plasma mass spectrometry with laser ablation. Analysis distinguished three formation periods: prenatal, early postnatal (to ~1 year), and early childhood.	The average manganese content was higher in the prenatal period than in the postnatal period.	Associations were found between intrusion errors and prenatal Mn levels, and between short- and long-delay recall and childhood Mn levels, particularly in males.
Savabieasfahani [47]	To investigate the relationship between increased military activity and increased metal content in primary teeth.	Primary teeth were collected from children from Iraq and Iran. The content of Li, Mg, Ca, Cr, Mn, Zn, Sr, Ba, Pb, Th and U was measured using laser ablation-inductively coupled plasma-mass spectrometry.	Pb was present in all dental tissues, with the highest levels in children with congenital defects. Higher amounts of Zn were found in dentin than in enamel. U and Th were not present in any samples.	Children were exposed to comparable amounts of Pb and other neurotoxic metals, often prenatally.
Fischer [48]	Analysis of Ca, Mn, Fe, Mg, Cu, K, Cr, Pb and Cd concentrations in hard tissues of primary teeth.	The primary teeth were collected from healthy children who had lived their entire lives in Silesia. The content of elements was examined using the atomic absorption spectroscopy.	The teeth contained the most Ca, K, Mg, and the least Cd, Mn, Cu, and Cr.	The concentration of metals in the tissues of primary teeth decreases with age.
Bayo et al. [49]	To assess the levels of lead and cadmium in shed deciduous teeth of children from Cartagena, Spain.	All 371 deciduous teeth were labeled, refrigerated in paper bags, then analyzed for lead and cadmium using microwave acid digestion and differential pulse anodic stripping voltammetry.	Higher lead and cadmium in children’s deciduous teeth were found in socially disadvantaged families and polluted areas. Lead increased with upper jaw teeth and thumb-sucking/nail-biting. Cadmium increased in non-carious teeth and without school fluoride. Both metals concentrated more in incisors than molars and decreased with tooth weight. No sex differences observed.	Children in Cartagena showed low lead and cadmium in deciduous teeth, affected by environmental and physiological factors, with tooth type being the primary determinant.
Bayo et al. [50]	To present the lead and cadmium levels measured in deciduous teeth of children from Cartagena, Spain, and examine their relationships with various variables.	A total of 834 shed, deciduous teeth were collected from children and subjected to analytical determination of lead and cadmium.	Lead distribution was log-normal while cadmium was not. No sex differences found. Metal levels decreased from incisors to molars and with age. Key environmental factors: fluoride use, residential zone, home age, and family socioeconomic status (latter two affecting lead).	Cartagena residents have low lead and cadmium levels, with lead influenced by tooth type, jaw, home age, and residential zone, while cadmium was affected only by zone of residence.
Dinca et al. [25]	To examine the impact of toxic metals on primary teeth and the influence of different drinks on the teeth dissolution and demineralization.	A total of 93 primary teeth from areas with varying pollution levels were analyzed for toxic metals using ICP-MS and tested for dissolution in sugary and sugar-free drinks. Teeth were weighed before and after immersion, with surfaces examined using AFM and SEM.	Teeth from industrial areas had higher toxic metal content, especially zinc. Carious teeth showed higher metal levels and greater material loss in sugary drinks, which caused more demineralization and surface roughness, particularly in carious teeth.	Teeth from polluted areas show higher toxic metal levels, more material loss in sugary drinks, and greater demineralization.
Motevaseliana et al. [52]	Association between lead (Pb) and cadmium (Cd) levels in primary teeth and saliva and dental caries in children living in Tehran, Iran.	Primary teeth from 211 Tehran children (6–11 years) were cleaned, stimulated saliva collected via paraffin chewing, pH measured with test strips, and Pb/Cd levels in teeth and saliva determined using atomic absorption spectrophotometry.	Mean concentrations in teeth: Pb 213.26 ppb, Cd 23.75 ppb; in saliva: Pb 11.83 ppb, Cd 3.18 ppb. Posterior teeth had higher Pb (235.20 ppb) than anterior teeth (107.87 ppb, *p* = 0.006). No age or gender differences in metal concentrations.	Despite finding no association between Pb or Cd levels and dental caries, there were high Pb and Cd concentrations in many children’s samples.
Johnston et al. [53]	To investigate historical lead (Pb) exposure near a lead-acid battery recycling facility in Vernon, California by analyzing deciduous teeth as a biological matrix reflecting toxic metal exposure.	Fifty deciduous teeth from 43 Latinx children were collected to measure pre/postnatal Pb exposure and analyzed in relation to soil lead data. Pb and As concentrations were determined using LA-ICP-MS.	Mean prenatal lead (208 Pb:43Ca ratio): 4.104 × 10^−4^ (SD 4.123 × 10^−4^); postnatal: 4.109 × 10^−4^ (SD 3.369 × 10^−4^); high correlation between periods (r = 0.87). Of 50 teeth, 20 showed prenatal arsenic and 17 postnatal arsenic. Females had higher lead levels and stronger soil-to-tooth lead associations than males. Children with detectable arsenic averaged 2.5 × 10^−4^ (95% CI 0.20, 4.80) higher prenatal lead levels.	Environmental lead contamination creates a cycle of exposure affecting both mothers and their developing children. Association between soil lead levels and prenatal tooth lead concentrations was found.

**Table 2:** Detailed characteristic of included studies.

Study	Study Location	Sample Type, N (Number of Samples)	Determination Method	Metals Analyzed	Levels of Concentration	Environmental Factors
Tvinnereim et al. [38]	19 counties of Norway, both urban and rural areas.	1271 deciduous teeth without fillings. Pb was determined in 1271 samples (139 incisors, including 21 with caries, 452 canines, including 45 with caries, and 680 molars, including 290 with caries), Hg in 554 (16 incisors, including 3 with caries, 152 canines, including 18 with caries, and 386 molars, including 174 with caries), Cd in 1235 (140 incisors, including 21 with caries, 442 canines, including 44 with caries, and 660 molars, including 281 with caries), and Zn in 1263 (140 incisors, including 21 with caries, 451 canines, including 45 with caries, and 238 molars, including 119 with caries).	Zn—the flame technique (FAAS), Pb and Cd—the graphite furnace technique (GFAAS), Hg—the cold vapor technique (CV-AAS).	Zn, Pb, Cd, Hg	Overall mean elemental content in deciduous teeth: 1.37 µg/g of Pb, 0.267 µg/g of Hg, 0.113 µg/g of Cd, 157 µg/g of Zn. Incisors: 1.45 µg/g of Pb, 0.06 µg/g of Hg, 0.04 µg/g of Cd, 141 µg/g of Zn. Canines: 1.17 µg/g of Pb, 0.06 µg/g of Hg, 0.04 µg/g of Cd, 134 µg/g of Zn. Molars: 1.11 µg/g of Pb, 1.10 µg/g of Hg, 0.005 µg/g of Cd, 149 µg/g of Zn. Carious: 1.36 µg/g of Pb, 0.13 µg/g of Hg, 0.05 µg/g of Cd, 169 µg/g of Zn. Non-carious: 1.10 µg/g of Pb, 0.07 µg/g of Hg, 0.04 µg/g of Cd, 133 µg/g of Zn. Carious incisors: 1.59 µg/g of Pb, 0.117 µg/g of Hg, 0.048 µg/g of Cd, 140 µg/g of Zn. Non-carious incisors: 1.42 µg/g of Pb, 0.056 µg/g of Hg, 0.043 µg/g of Cd, 141 µg/g of Zn. Carious canines: 1.41 µg/g of Pb, 0.099 µg/g of Hg, 0.039 µg/g of Cd, 136 µg/g of Zn. Non-carious canines: 1.15 µg/g of Pb, 0.060 µg/g of Hg, 0.039 µg/g of Cd, 133 µg/g of Zn. Carious molars: 1.34 µg/g of Pb, 0.136 µg/g of Hg, 0.047 µg/g of Cd, 177 µg/g of Zn. Non-carious molars: 0.97 µg/g of Pb, 0.079 µg/g of Hg, 0.044 µg/g of Cd, 131 µg/g of Zn. Roots: 1.31 µg/g of Pb, 0.09 µg/g of Hg, 0.05 µg/g of Cd, 153 µg/g of Zn. No roots: 1.00 µg/g of Pb, 0.09 µg/g of Hg, 0.04 µg/g of Cd, 129 µg/g of Zn.	Presence of caries.
Nazemisalman et al. [39]	Zanjan, Iran.	Forty-two deciduous teeth, divided into tooth type (incisors, molars), location (upper, lower.)	Polarographic system based on anodic stripping voltammetry (ASV).	Zn, Cd, Pb, Cu.	Overall mean elemental content: 7.66 µg/g of Pb, 5.33 µg/g of Cu, 0.0879 µg/g of Cd, 245 µg/g of Zn. Female: 8.20 µg/g of Pb, 5.91 µg/g of Cu, 0.08 µg/g of Cd, 293.16 µg/g of Zn. Male: 6.91 µg/g of Pb, 4.56 µg/g of Cu, 0.09 µg/g of Cd, 181.37 µg/g of Zn. From a donor in the age range of 1–5 years: 6.33 µg/g of Pb, 4.90 µg/g of Cu, 0.08 µg/g of Cd, 224.48 µg/g of Zn. from a donor in the age range of 5–10 years: 8.88 µg/g of Pb, 5.73 µg/g of Cu, 0.09 µg/g of Cd, 264.13 µg/g of Zn. Incisors: 8.98 µg/g of Pb, 5.02 µg/g of Cu, 0.09 µg/g of Cd, 311.32 µg/g of Zn. Molars: 7.20 µg/g of Pb, 5.44 µg/g of Cu, 0.08 µg/g of Cd, 221.80 µg/g of Zn. Upper teeth: 6.79 µg/g of Pb, 3.88 µg/g of Cu, 0.06 µg/g of Cd, 107.09 µg/g of Zn. Lower teeth: 8.02 µg/g of Pb, 5.91 µg/g of Cu, 0.10 µg/g of Cd, 214.93 µg/g of Zn.	Urban environment far from factories; high socio-economic status.
Tacail et al. [40]	No data.	Twelve permanent molars and ten deciduous teeth (4 incisors, 2 canines and 4 molars), longitudinally sectioned along the buccal–lingual axis.	Laser ablation inductively coupled plasma mass spectrometry (LA-ICP MS).	Ca, Cu, Zn, Ni, Sr, Ba, Pb.	No data.	No data.
Adams et al. [37]	Arizona State, USA.	Teeth obtained from 15 children on the autism spectrum and 11 neurotypical controls.	Hg—Cold vapor atomic absorption spectrophotometry (CV-AAS), Zn—flame atomic absorption spectrophotometer (flame AA), Pb—graphite furnace atomic absorption spectrophotometer (GFAAS).	Hg, Pb, Zn.	Mean of elements: Hg: Autism: 0.15 ± 0.11 μg/g Control: 0.07 ± 0.06 μg/g Pb: Autism: 0.38 ± 0.32 μg/g Control: 0.29 ± 0.14 μg/g Zn: Autism: 100 ± 20 μg/g Control: 98 ± 16 μg/g	Maternal fish consumption during pregnancy; Presence or placement of amalgam fillings in the mother during pregnancy; Presence of amalgam fillings in the child. Administration of anti-D immunoglobulin (Anti-Rho D) to the mother; Maternal use of antibiotics during pregnancy and antibiotic use in the child up to 48 months of age; Paint consumption (pica behavior).
Ericson [56]	Los Angeles, California, USA (urban area), Tijuana, Mexico (urban area) Sinaloa, Mexico (rural area).	Three longitudinal sections through the enamel of exfoliated primary mandibular central incisors.	Secondary-ion mass spectrometry (SIMS).	Pb, Ca.	Total Pb concentration: Los Angeles—1.1 ppm Tijuana—3.7 ppm Sinaloa—1.57 ppm Ca—no numeric data	Area of residence (urban/rural); Exposure to tableware covered with lead glaze; Consumption of lead-contaminated sweets; Presence of car batteries in the residence.
Nedelescu [41]	Industrial area Copsa Mica, Sibiu County, Romania.	Entire primary teeth-after physiological resorption; hair (no numbers of samples for either samples).	Inductively coupled plasma mass spectrometry (ICP-MS).	Cd, Pb, Cu, Zn, Cr, Mn, Co, Ni.	Tooth samples from polluted area (μg/g): Cd—0.018 Pb—0.77 Cu—0.74 Zn—52.70 Cr—11.73 Mn—1.99 Co—0.98 Ni—46.16 Tooth samples from unpolluted area (μg/g): Cd—0.012 Pb—0.08 Cu—0.52 Zn—61.51 Cr—8.33 Mn—0.43 Co—0.52 Ni—23.52	Activity of smelter.
Sitarik [55]	Detroit, Michigan.	Enamel and dentine of primary teeth (*N* = 146).	Laser ablation inductively coupled plasma mass spectrometry (LA-ICP-MS).	Pb.	Suburban: 2nd trimester: 0.021 (0.026) 3rd trimester: 0.027 (0.030) Postnatal: 0.022 (0.028) 0.003 Urban: 2nd trimester: 0.028 (0.039) 3rd trimester: 0.041 (0.056) Postnatal: 0.033 (0.027)	Location of residence; Environmental tobacco smoke during pregnancy; Mother smoked during pregnancy; Prenatal indoor pets; Year house was built; Prenatal antibiotic use; Prenatal antifungal use; Season of birth.
Amr [42]	El-Kanayat City, Egypt.	Sixty-four primary teeth pulp.	Inductively coupled plasma mass spectrometry (ICP-MS).	Na, Mg, Al, Cr, Zn, Ag, Fe, Ni, Cu, Sr, Cd, Ba, Pb, U, Mn, Co, As, Se, Mo, Bi.	Na 5454 ± 950 ppm Mg 1755 ± 340 ppm Al 17.9 ± 12.3 ppm Cr 0.04 ± 0.01 ppm Mn 5.5 ± 2 ppm Co 0.54 ± 0.12 ppm Fe 80.1 ± 16.5 ppm Ni 1.66 ± 0.46 ppm Cu 6.4 ± 4.8 ppm Zn 133 ± 30 ppm As 0.82 ± 0.07 ppm Se 10.5 ± 1.57 ppm Sr 87 ± 11.3 ppm Mo 1.8 ± 0.29 ppm Ag 0.08 ± 0.03 ppm Cd 0.00011 ± 0.00001 ppm Ba 7.8 ± 3.2 ppm Pb 1.2 ± 0.89 ppm Bi 23 ± 2.34 ppm U 0.005 ± 0.002 ppm	No data.
Gomes [43]	Piracicaba, Brazil.	Enamel of primary teeth (*N* = 329).	Graphite furnace atomic absorption spectrometry (GFAAS).	Pb.	Non-industrial 183.1 (190.6) μg /g Industrial 275.5 (326.7) μg /g	Industrial area.
Abdullah [44]	No data.	Enamel of primary teeth (*N* = 84).	Laser ablation inductively coupled plasma mass spectrometry (LA-ICP-MS).	Pb, Hg, Mn.	Prenatal Pb ASD = 0.27 (0.27) ppm TD = 0.38 (0.59) ppm HDB = 0.33 (0.33) ppm TD = 0.32 (0.36) ppm Postnatal Pb ASD = 0.29 (0.29) ppm TD = 0.43 (0.61) ppm HDB = 1.10 (3.47) ppm TD = 0.22 (0.23) ppm Prenatal Hg ASD = 1.42 (0.61) ppm TD = 1.90 (2.79) ppm HDB = 1.27 (0.76) ppm TD = 1.82 (2.34) ppm Postnatal Hg ASD = 1.47 (0.77) ppm TD = 1.45 (0.90) ppm HDB = 1.01 (0.45) ppm TD = 1.22 (0.70) ppm Prenatal Mn ASD = 1.41 (1.10) ppm TD = 1.63 (0.95) ppm HDB = 1.62 (0.77) ppm TD = 1.58 (0.88) ppm Postnatal Mn ASD = 1.87 (2.01) ppm TD = 2.91 (2.43) ppm HDB = 2.11 (2.22) ppm TD = 1.80 (1.70) ppm	No data.
Rosa [45]	Mexico City, Mexico.	Dentin of primary teeth (*N* = 291).	Laser ablation inductively coupled plasma mass spectrometry (LA-ICP-MS).	As, Cd, Co, Cu, Mn, Ni, Pb.	Mn = 0.41 Cd = 0.30 Pb = 0.17 As = 0.09 Co = 0.02 Ni = 0.01 Cu = 0.00	No data.
Yalcin [46]	Ankara, Turkey.	Dentin of primary teeth (*N* = 25).	Inductively coupled plasma mass spectrometry (ICP-MS).	Ca, Mg, Zn, Cd, Hg, Pb, Cu, Cr, Fe, Mn, Mo, P, Sr.	Mg, mg/g TDC = 6.1 NDD = 5.5 P, mg/g TDC = 143 NDD = 129 Ca, mg/g TDC = 251 NDD = 248 Cr, μg/g TDC = 0.06 NDD = 0.07 Mn, μg/g TDC = 1.02 NDD = 0.58 Fe, μg/g TDC = 4.05 NDD = 3.77 Cu, μg/g TDC = 0.12 NDD = 0.24 Zn, μg/g TDC = 106 NDD = 101 Sr, μg/g TDC = 95.2 NDD = 54.2 Mo, μg/g TDC = 0.02 NDD = 0.03 Cd, μg/g TDC = 0.002 NDD = 0.002 Pb, μg/g TDC = 0.57 NDD = 0.68 Hg, μg/g TDC and NDD < DL	Smoking exposure.
Friedman [54]	Bagnolo Mella, Valcamonica and Garda Lake, Italy.	Dentin of primary teeth (*N* = 280).	Laser ablation inductively coupled plasma mass spectrometry (LA-ICP-MS).	Mn.	Patients with intrusion errors: Prenatal = 0.23 Postnatal = 1.16 Childhood = 1.09 Patients with perseveration errors: Prenatal = 1.50 Postnatal = 0.92 Childhood = 1.00	Active/historical/no history of ferroalloy production.
Savabieasfahani [47]	Iraq, Iran, Lebanon.	Dentin and enamel of primary teeth (*N* = 9).	Laser ablation inductively coupled plasma mass spectrometry (LA-ICP-MS).	Li, Mg, Ca, Cr, Mn, Zn, Sr, Ba, Pb, Th, U.	No data	War zone.
Fischer [48]	Silesia, Poland.	Primary teeth (*N* = 45).	Flame atomic absorption spectrometry (FAAS).	Ca, Mn, Fe, Mg, Cu, K, Cr, Pb and Cd.	Mn 4.39 μg/g Fe 51.0 μg/g Pb 13.4 μg/g Cd 0.70 μg/g Cu 4.62 μg/g Cr 8.97 μg/g K 240 μg/g Ca 20.0 μg/g Mg 2367 μg/g	No data.
Bayo et al. [49]	Three zones in Cartagena, Spain- nonpolluted zone, polluted zone, intermediate zone.	Three hundred and seventy-one deciduous teeth; entire incisors, canines and molars.	Differential pulse anodic stripping voltammetry (DPASV).	Pb, Cd.	Pb (~3.5 μg/g): Higher in upper jaw, non-carious teeth, thumb-sucking/nail-biting habits, disadvantaged families, polluted areas, smoking fathers, no school fluoride. Decreases from incisors to molars. Cd (~58 ng/g): Higher in non-carious teeth, polluted zones, older houses, no school fluoride. Decreases from incisors to molars.	Home antiquity, socioeconomic status, parents’ smoking habit, thumb-sucking and nail-biting habits, pollution of the residential zone, use of fluoride.
Bayo et al. [50]	Cartagena, Spain.	Eight hundred and thirty-four shed, deciduous teeth.	Differential pulse anodic stripping voltammetry (DPASV).	Pb and Cd.	Cd: 59.776 ± 63.68 μg/g; lower in older children, with fluoride use, in upper jaw, and in incisors. Pb: 4.17 ± 2.64 μg/g; lower in newer houses, older children, higher socioeconomic status, non-smoking fathers, school fluoride use, less-polluted areas, upper jaw, and incisors.	Home antiquity, family socio-economic status, parents’ smoking habit, thumb-sucking habit, zone of residence, use of fluoride.
Dinca et al. [25]	Bucharest, Romania Pătârlagele city as a less polluted city and Slatina city as more polluted.	Ninety-three deciduous teeth; carious and non-carious.	Inductively coupled plasma mass spectrometry (ICP-MS).	Cu, Pb, Cd, Cr, Zn.	Cd: Pătârlagele: Carious: 0.06 ppb Non-carious: 0.04 ppb Slatina: Carious: 0.13 ppb Non-carious: 0.08 ppb Pb: Pătârlagele: Carious: 0.05 ppb Non-carious: 0.02 ppb Slatina: Carious: 0.075 ppb Non-carious: 0.063 ppb Cr: Pătârlagele: Carious: 1.22 ppb Non-carious: 0.8 ppb Slatina: Carious: 1.27 ppb Non-carious: 1.10 ppb Cu: Pătârlagele: Carious: 0.3 ppb Non-carious: 0.08 ppb Slatina: Carious: 0.72 ppb Non-carious: 0.41 ppb Zn: Pătârlagele: Carious: 221.36 ppb Non-carious: 435.78 ppb Slatina: Carious: 199.44 ppb Non-carious: 385.60 ppb	Air pollution.
Motevaseliana et al. [52]	Tehran, Iran.	Anterior and posterior deciduous teeth with and without caries; *N* = 211.	Graphite furnace atomic absorption spectrometry (GFAAS).	Pb, Cd.	Teeth: - Pb: 213.26 ppb (95% CI: 164.29–274.84 ppb) - Cd: 23.75 ppb (95% CI: 20.86–27.05 ppb) Saliva: - Pb: 11.83 ppb (95% CI: 10.71–13.06 ppb) - Cd: 3.18 ppb (95% CI: 2.69–3.75 ppb)	- Socioeconomic status (family income, parental education, housing status); - Region of residence within Tehran (affluent vs. non-affluent areas); - Oral hygiene behaviors (tooth brushing frequency, dental visits); - Dietary habits (snacking frequency); - Passive smoking; - Salivary pH.

**Table 3:** Quality assessment—JBI checklist for quasi-experimental studies (nonrandomized experimental studies).

Authors	1. Is It Clear in the Study What Is the “Cause” and What Is the “Effect”?	2. Were the Participants Included in Any Similar Comparisons?	3. Were the Participants Included in Any Comparisons Receiving Similar Treatment/Care, Other than the Exposure or Intervention of Interest?	4. Was There a Control Group?	5. Were There Multiple Measurements of the Outcome Both Pre- and Post- Intervention/ Exposure?	6. Was Follow Up Complete and If Not, Were Differences Between Groups in Terms of Their Follow Up Adequately Described and Analyzed?	7. Were the Outcomes of Participants Included in Any Comparisons Measured in the Same Way?	8. Were Outcomes Measured in a Reliable Way?	9. Was Appropriate Statistical Analysis Used?
Tvinnereim [38]	Yes	Yes	Yes	Yes	No	Yes	Yes	Yes	Yes
Nazemisalman [39]	Yes	Yes	Yes	No	No	Yes	Yes	Yes	Yes
Tacail [40]	Yes	Yes	Yes	No	No	Yes	Yes	Yes	No
Adams [37]	Yes	Yes	Yes	Yes	No	Yes	Yes	Yes	Yes
Ericson [56]	Yes	Yes	Yes	No	No	Yes	Yes	Yes	No
Nedelescu [41]	Yes	Yes	Yes	Yes	No	Yes	Yes	Yes	Yes
Sitarik [55]	Yes	Yes	Yes	No	No	Yes	Yes	Yes	Yes
Amr [42]	Yes	Yes	Yes	No	No	Yes	Yes	Yes	Yes
Gomes [43]	Yes	Yes	Yes	Yes	No	Yes	Yes	Yes	Yes
Abdullah [44]	Yes	Yes	Yes	No	No	Yes	Yes	Yes	Yes
Rosa [45]	Yes	Yes	Yes	No	No	Yes	Yes	Yes	Yes
Yalcin [46]	Yes	Yes	Yes	Yes	No	Yes	Yes	Yes	Yes
Friedman [54]	Yes	Yes	Yes	No	No	Yes	Yes	Yes	Yes
Savabieasfahani [47]	Yes	Yes	Yes	No	No	Yes	Yes	Yes	No
Fischer [48]	Yes	Yes	Yes	No	No	Yes	Yes	Yes	Yes
Bayo [49]	Yes	Yes	Yes	No	No	Yes	Yes	Yes	Yes
Bayo [50]	Yes	Yes	Yes	No	No	Yes	Yes	Yes	Yes
Dinca [25]	Yes	Yes	Yes	Yes	No	Yes	Yes	Yes	Yes
Motevaseliana [52]	Yes	Yes	Yes	No	No	Yes	Yes	Yes	Yes
Johnston [53]	Yes	Yes	Yes	No	Yes	Yes	Yes	Yes	Yes

## Data Availability

Data supporting the findings of this study are available within the article.
